# The Expression of Tristetraprolin and Its Relationship with Urinary Proteins in Patients with Diabetic Nephropathy

**DOI:** 10.1371/journal.pone.0141471

**Published:** 2015-10-30

**Authors:** Fengxun Liu, Jia Guo, Qian Zhang, Dongwei Liu, Lu Wen, Yang Yang, Liu Yang, Zhangsuo Liu

**Affiliations:** 1 Department of Nephrology, The First Affiliated Hospital of Zhengzhou University, Zhengzhou, People’s Republic of China; 2 Nephropathy Research Institutes of Zhengzhou University, Zhengzhou, People’s Republic of China; 3 Key-Disciplines Laboratory Clinical-Medicine Henan, Zhengzhou, People’s Republic of China; Emory University, UNITED STATES

## Abstract

**Objective:**

Tristetraprolin (TTP), also known as zinc finger protein 36, is an RNA binding protein that has a significant role in regulating the expression of mRNAs containing AU-rich elements. We postulated that TTP might regulate interleukin (IL)-6 and IL-18 expression in diabetes. This study aimed to test the hypothesis that the levels of TTP are correlated with nephropathy in patients with type 2 diabetes.

**Methods:**

Eighty-seven patients (61.3±9.6 years old) who had been diagnosed with type 2 diabetes mellitus and 41 age and sex matched healthy control subjects were enrolled. The diabetes patients were classified into those without proteinuria, with microalbuminuria, and with clinical proteinuria groups according to the ratio of urinary excretion of albumin/creatinine (ACR).

**Results:**

Serum and urinary levels of IL-6 and IL-18 were significantly elevated, but those of TTP were significantly decreased in patients with diabetes as compared with control subjects. In addition, serum and urinary levels of IL-6 and IL-18 were significantly higher, but those of TTP were significantly lower in patients with proteinuria than in patients without proteinuria or with microalbuminuria. There was a significant correlation between serum TTP and IL-6/IL-18 (correlation coefficients of -0.572 and -0.685, *P* < 0.05).

**Conclusion:**

These results show that diabetes with clinical proteinuria is accompanied by decreased urinary and serum level of TTP and increased levels of IL-6 and IL-18. Decreased TTP expression might occur prior to the increase in IL-6 and IL-18, and decrease of TTP might provide an earlier marker for glomerular dysfunction than IL-6 and IL-18.

## Introduction

Diabetes mellitus has become the leading cause of chronic renal failure in developed countries and is increasing as a cause of morbidity and mortality worldwide [1]. Diabetic nephropathy (DN) is one of the most common microvascular complications of diabetes mellitus and has become the principal cause of end-stage renal failure. About 31% -40% of diabetes mellitus patients develop diabetic nephropathy, which poses a serious health threat [2].

Inflammation is a key pathophysiological mechanism of diabetic nephropathy, for which inflammatory molecules and mediators are important in the early stage. The potential clinical use of inflammatory markers, such as IL-1, IL-6, IL-18 and TNF, as predictors of DN is under consideration. The serum levels of IL-6, which is directly associated with early glomerular structural abnormalities, have been shown to be substantially higher in type 2 diabetes mellitus (T2DM) patients with DN than in T2DM patients without renal disease. Given the association of IL-18 with multiple related inflammatory diseases, the levels of IL-18 also might be useful as an early marker of renal dysfunction in patients with T2DM [3,4].

Tristetraprolin (TTP) is a well-characterized zinc finger-containing RNA-binding protein. TTP binds to and destabilizes mRNAs with 3′untranslated regions that contain AU-rich elements (AREs), including those of CCL2, IL-6, IL-10, TNF-α, and many other inflammatory factors. Consequently, TTP functions as an anti-inflammatory protein and is involved in many different pathological and physiological processes. However, the relationship of TTP with inflammatory cytokines in diabetic patients is still unknown. Additionally, it remains unknown whether levels of TTP might serve as early markers of renal dysfunction in diabetic patients [5,6].

We performed this study to determine the expression levels of TTP in human blood and urine and to analyze the relationship between serum TTP level and levels of the IL-6 and IL-18 in diabetic patients. These results provide evidence that TTP might serve as a new early marker, and potentially a therapeutic target, for renal dysfunction in diabetic patients.

## Material and Methods

### 2.1 Patients

Eighty-seven patients from the First Affiliated Hospital of Zhengzhou University (61.3±9.6 years old) who had been diagnosed with T2DM (with or without albuminuria) were enrolled between March 2013 and February 2014. These patients had fasting blood glucose greater than 7.0 mmol / L, 2-hour postprandial blood glucose greater than 11.1 mmol / L and no acute complications. Patients with the following disease complications were excluded: primary acute and chronic glomerulonephritis; renal artery stenosis; severe malnutrition; acute cardiovascular or cerebrovascular diseases; secondary glomerular disease as determined by renal biopsy; autoimmune diseases; infectious diseases. Patients with three month history of blood transfusion or use of analgesics, corticosteroids, or immunosuppressants were also excluded.

The enrolled patients were divided into three groups according to the ratio of urinary excretion of albumin to creatinine (ACR): diabetic patients without proteinuria (ACR: less than 30 mg/g, n = 33), with microalbuminuria (ACR: 30 to 300 mg/g, n = 29), and with clinical proteinuria (ACR: more than 300 mg/g, n = 25).

In addition, 41 normal healthy control subjects were recruited from those who attended to our hospital to have physical examination and who were confirmed to be normotensive and without diabetes mellitus, kidney disease or other serious diseases. None of the control subjects were detected urine proteins or had ACR>30 mg/g creatinine.

Urine and blood samples were collected from diabetes patients at the time of a clinical visit and from normal healthy control subjects upon the physical examination. Three ml venous blood was collected into the dry tubes, centrifuged, and the supernatant was collected for analysis. In addition, first voided midstream urine samples were collected in tubes containing RNAase inhibitors. The urine samples were centrifuged, and the supernatant was collected for analysis. Written informed consent was obtained from all study participants prior to sample collection. The study was approved by the Ethical Committee of the First Affiliated Hospital of Zhengzhou University.

### 2.2 Reverse transcriptase polymerase chain reaction (RT-PCR)

Blood and urine samples were centrifuged at 1000 g for 10 minutes and supernatants were collected. cDNA was synthesized using reverse transcriptase (Thermo Fisher Scientific Massachusetts USA). PCR was performed according to the manufacturer’s instructions (Thermo Fisher Scientific Massachusetts USA). The PCR conditions were as shown in Table 1. All reactions were run using a certified PCR instrument (Veriti, Life Technologies /AB & Invitrogen, California, USA). PCR products were separated on polyacrylamide gels (Novex TBE gels 6% Cat. No. EC6265BOX, Invitrogen), stained with SYBR-safe DNA gel stain (Cat. no. S33102, Invitrogen) and photographed under UV light using a G-BOX (Syngene, Cambridge, UK). The blots in gel were scanned the grayscale using Image J software, and the target gene expression was calculated as the grayscales normalized to those of β-actin gene. The nucleotide sequences of the PCR primers were as follows: TTP (241bp): 5’-GGAGTGTCTTCCGAGGTTCTT-3’(sense) and 5’GCTACTTGCTTTTGGAGGGTAAT3’(antisense);IL-18(185bp):5’-ACAGTCAGCAAGGAATTGTCTC-3’(sense) and 5’GGTTCAGCAGCCATCTTTATTC3’(antisense);IL-6(150bp):5’-AACCTTCCAAAGATGGCTGAA-3’(sense) and 5’GGCTTGTTCCTCACTACTCTCAA-3’ (antisense);β-actin(285bp):5’-AGCGAGCATCCCCCAAATT-3’(sense) and 5’GGGCACGAAGGCTCATCATT-3’(antisense).PCR reaction conditions were showed in Table 1.

**Table 1:** PCR reaction conditions.

	Indicators	Temperature	Time	
	Pre-denaturation	94°C	3 mins	
	Denaturation	94°C	30 s	
β-actin	Anneal	60°C	30 s	33 Cycles
	Extension	72°C	1 min	
	Extension	72°C	5 mins	
	End	4°C	pause	
	Pre-denaturation	94°C	3 mins	
	Denaturation	94°C	30 s	
IL-6	Anneal	50°C	30 s	35 Cycles
	Extension	72°C	1 min	
	Extension	72°C	5 mins	
	End	4°C	pause	
	Pre-denaturation	94°C	3 mins	
	Denaturation	94°C	30 s	
IL-18	Anneal	59°C	30 s	30 Cycles
	Extension	72°C	1 min	
	Extension	72°C	5 mins	
	End	4°C	pause	
	Predenaturation	94°C	3 mins	
	Denaturation	94°C	30 s	
TTP	Anneal	50°C	30 s	35 Cycles
	Extension	72°C	1 min	
	Extension	72°C	5 mins	
	End	4°C	pause	

### 2.3 ELISA analysis and biochemical investigations

Blood samples were centrifuged at 1000 g for 10 minutes. Serum specimens were then frozen and stored at −80°C until analysis. The serum levels of human IL-6 and IL-18 (Sangon Biotech, Shanghai China) and TTP (CUSABIO, Wuhan China) were measured using commercially available ELISA kits.

High-sensitive C-reactive protein (hsCRP), creatinine (Cr), blood urea nitrogen (BUN), hemoglobin A1c (HbA1c), triglyceride (TG), and albumin were measured using an automatic biochemical analyzer, and the ACR (albumin to creatinine ratio) was determined based on these values.

### 2.4 Statistical analysis

All values were analyzed using statistical software SPSS17.0 and presented as means ± standard error for continuous variables. Analysis of variance and the following independent t tests were used for comparisons. Correlations between two variables were estimated by Spearman’s rank sum test. A p value of <0.05 was considered statistically significant.

## Results

### 3.1 Characteristics of study subjects

Eighty seven diabetes patients and 41 normal control subjects were enrolled in this study. The patient characteristics and the results of biochemical urine assays are shown in Table 2. There was no significant difference in age, gender, smoking and drinking among the 4 groups (P>0.05). All the control subjects did not take any drugs, while the diabetes patients routinely took insulin and oral drugs (including hypoglycemia drugs, angiotensin-converting enzyme inhibitor/Angiotensin Ⅱ receptor antagonist, statins). Diabetic patients were classified according to the level of proteinuria as defined by the ACR: diabetic patients without proteinuria (25.7±4.4 mg/g Cr), diabetic patients with microalbuminuria (99.2±10.5 mg/g Cr) or diabetic patients with clinical proteinuria (1023.8±280.9 mg/g Cr). The ACR of the normal control group was 20.5±9.6 mg/g Cr.

**Table 2:** Characteristics of all subjects.

	Normal healthy controls(41)	Diabetic patients without proteinuria (33)	Diabetic patients with microalbuminuria (29)	Diabetic patients with clinical proteinuria (25)	χ^2^	P
Age (yr)	60.5±8.9	59.3±11.4	61.7±9.4	62.3±11.0	0.5129	0.6741
Gender (M/F)	27/14	20/13	18/11	13/12	1.27	0.735
BMI (kg/m^2^)	25.1±0.5	27.2±0.4*	27.5±0.6	28.1±0.5	238.6	<0.001
HbA1c (%)	4.9±0.7	7.8±0.3*	7.5±0.8	8.4±0.4	240.5	<0.001
TG (mmol/L)	1.32±0.33	2.57±0.85*	2.96±0.64	3.02±1.16	38.93	<0.001
Cr (μmol/L)	75.47±11.97	77.30±15.78	74.77±17.14	80.86±13.24	0.9683	0.41
BUN (mmol/L)	5.21±1.37	5.77±0.84	4.86±1.04	6.05±1.13	6.469	0.001
ACR (mg/g)	20.5±9.6	25.7±4.4	99.2±10.5*	1023.8±280.9***	422.3	<0.001
H-CRP (mg/L)	0.81±0.44	2.69±1.53*	5.37±2.09*	12.19±3.74***	165.6	<0.001
Insulin (n/%)	0	28(84.8%)	26(89.7%)	23(92.0%)	91.40	<0.001
Hypoglycemia drugs (n/%)	0	23(69.7%)	24(82.8%)	22(88.0%)	72.67	<0.001
ACEI/ARB (n/%)	0	18(54.5%)	26(89.7%)	24(96.0%)	80.49	<0.001
Statins (n/%)	0	11(33.3%)	14(48.3%)	19(76.0%)	43.18	<0.001
Smoking (n/%)	12(29.3%)	14(42.3%)	11(37.9%)	8(32.0%)	1.59	0.660
Drink (n/%)	25(61.0%)	17(58.6%)	15(51.7%)	12(48.0%)	1.31	0.727
Complication						
Amputation (n/%)	0	0	1(3.5%)	0	3.441	0.329
Diabetic foot (n/%)	0	2(6.1%)	1(3.5%)	1(4.0%)	2.34	0.506
Peripheral circulation diseases (n/%)	0	4(12.1%)	3(10.3%)	6(24.0%)	10.04	0.018

The data are presented as mean±S.E.

*P < 0.05

***P < 0.001 versus normal healthy controls.

BMI: body mass index; HbA1c: hemoglobin A1c; TG: triglyceride; Cr: creatinine; BUN: blood urea nitrogen; ACR, albumin to creatinine ratio; H-CRP: high-sensitive C-reactive protein. ACEI: angiotensin-converting enzyme inhibitor; ARB: angiotensin Ⅱ receptor antagonist

### 3.2 Assessment of TTP/IL-6/ IL-18 mRNA and protein levels

**Fig 1:**
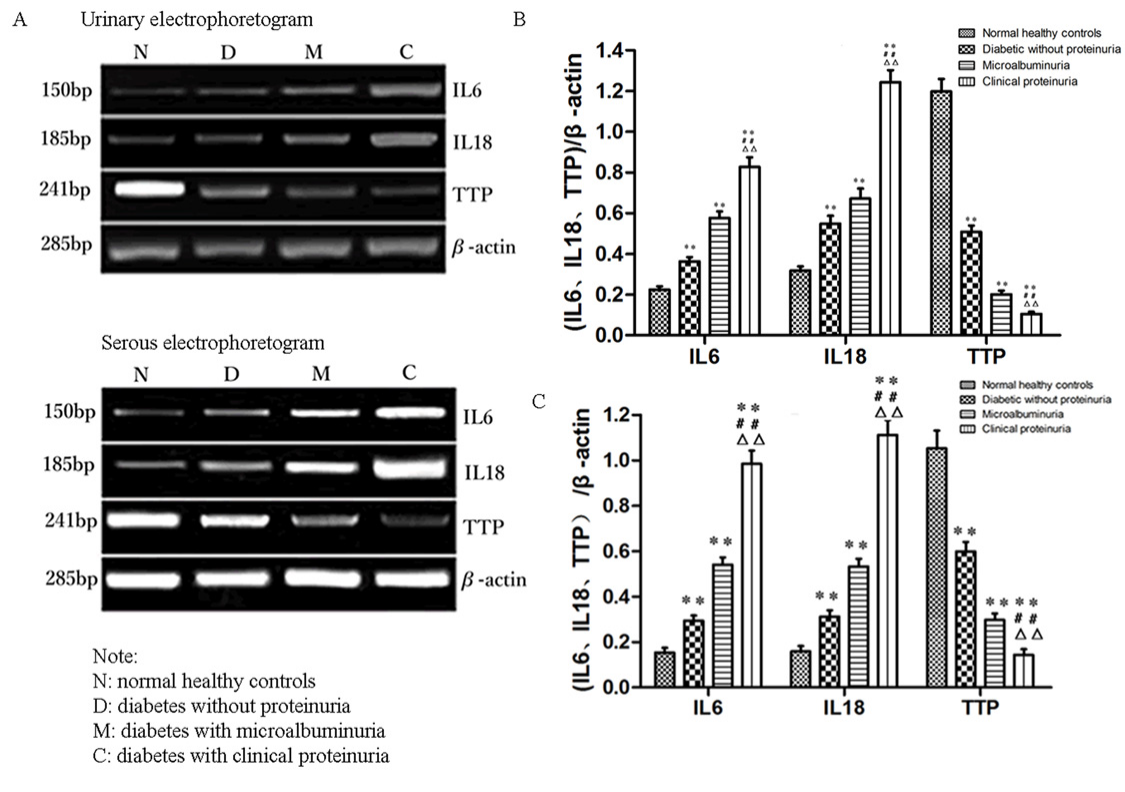
mRNA levels of IL-6, IL-18, and TTP in urine and serum samples. Samples from urine (A and B) and serum (A and C) were analyzed by semi-quantitative RT-PCR and visualized by electrophoresis on acrylamide gels. Representative gels are shown in A; and quantification of the results of patients from each group (normal, n = 41; diabetic without proteinuria, n = 33; diabetic with microalbuminuria, n = 29; and diabetic with clinical proteinuria, n = 25) is shown in B and C. The data are presented as mean±S.E. *P < 0.05, **P < 0.01 compared with normal healthy controls, ^##^ P<0.01 compared with diabetic without proteinuria, and ^△△^P<0.01 compared with diabetic with microalbuminuria.

To determine the relationship between the serum TTP level and levels of IL-6 and IL-18, we first conducted a PCR assay of urine and serum specimens. Urinary and serum levels of IL-6 and IL-18 mRNA were significantly elevated but those of TTP were significantly decreased in diabetes patients compared with healthy controls (P<0.001). In addition, urinary and serum levels of IL-6 and IL-18 mRNA were significantly increased but those of TTP were significantly decreased in patients with clinical proteinuria when compared with those without proteinuria or with microalbuminuria (P<0.001) (Fig 1). These results suggest that a decrease in TTP expression might occur in diabetic patients prior to the development of proteinuria and that the extent of the decrease becomes progressively larger as the proteinuria develops.

**Fig 2:**
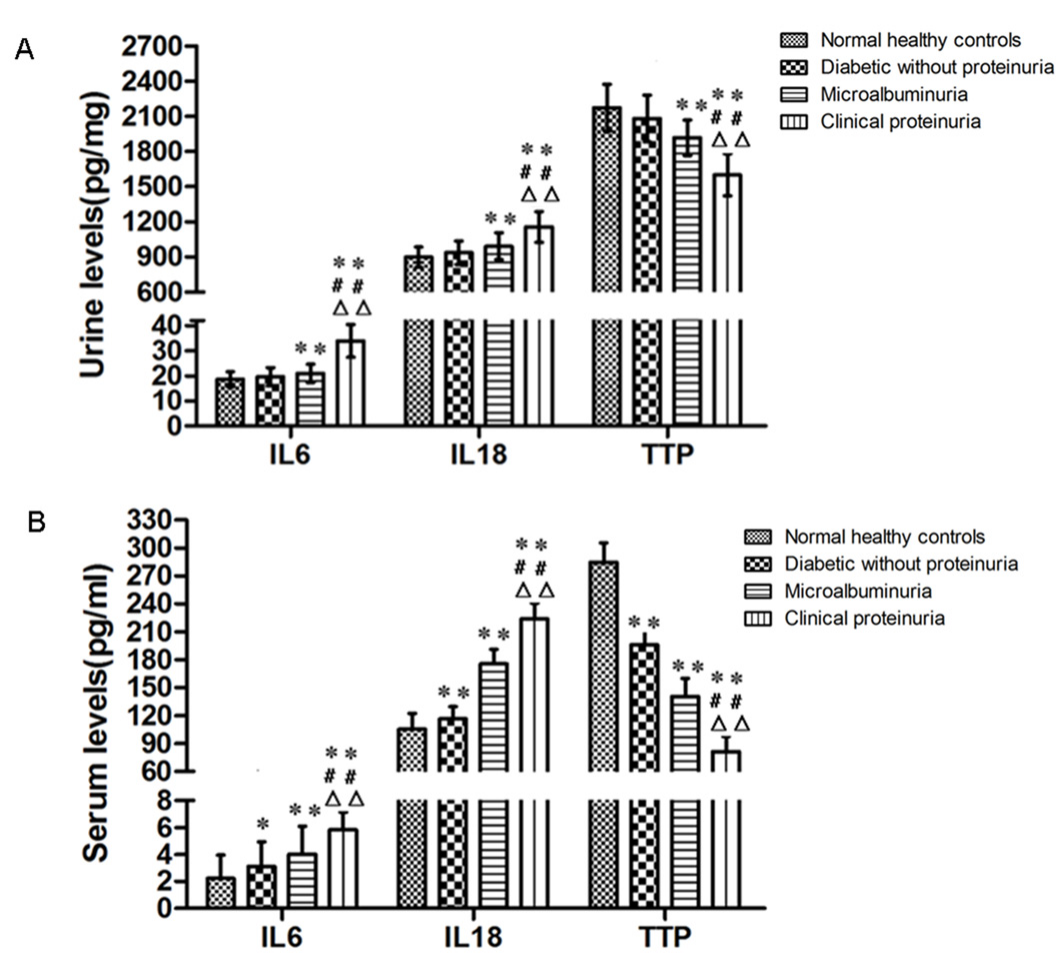
Protein contents of IL-6, IL-18, and TTP in urine and serum samples. Samples from urine (A) and serum (B) of patients from the normal (n = 41), diabetic without proteinuria (n = 33), diabetic with microalbuminuria (n = 29), and diabetic with clinical proteinuria (n = 25) groups were analyzed by ELISA. The data are presented as mean±S.E. *P< 0.05, **P < 0.01 compared with normal healthy controls, ^##^ P<0.01 compared with diabetic without proteinuria, and ^△△^P<0.01 compared with diabetic with microalbuminuria.

To verify the mRNA results and to determine whether the observed patterns of TTP, IL-6 and IL-18 mRNA expression could be detected at the protein level, we performed ELISA (Fig 2). Consistent with the mRNA data, a significant increase in IL-6 and IL-18 protein levels of urine and serum was observed in patients with microalbuminuria or clinical proteinuria when compared with healthy controls (P<0.001), and in patients with clinical proteinuria when compared with those without proteinuria or with microalbuminuria (P<0.001). However, this difference was less obvious at the earlier stages of DN for diabetic patients without proteinuria. Additionally, TTP protein expression was significantly decreased in diabetic with clinical proteinuria when compared with healthy controls or patients of other two groups (P<0.001). Because TTP is known to regulate the expression of multiple cytokines via its ability to bind to AREs [5,6], the expression of TTP at an early stage of disease is consistent with the possibility that decreased TTP expression precedes and regulates increased IL-18 and IL-6 in diabetes.

### 3.3 Serum level of TTP correlates inversely with the levels of IL-6 and IL-18

**Fig 3:**
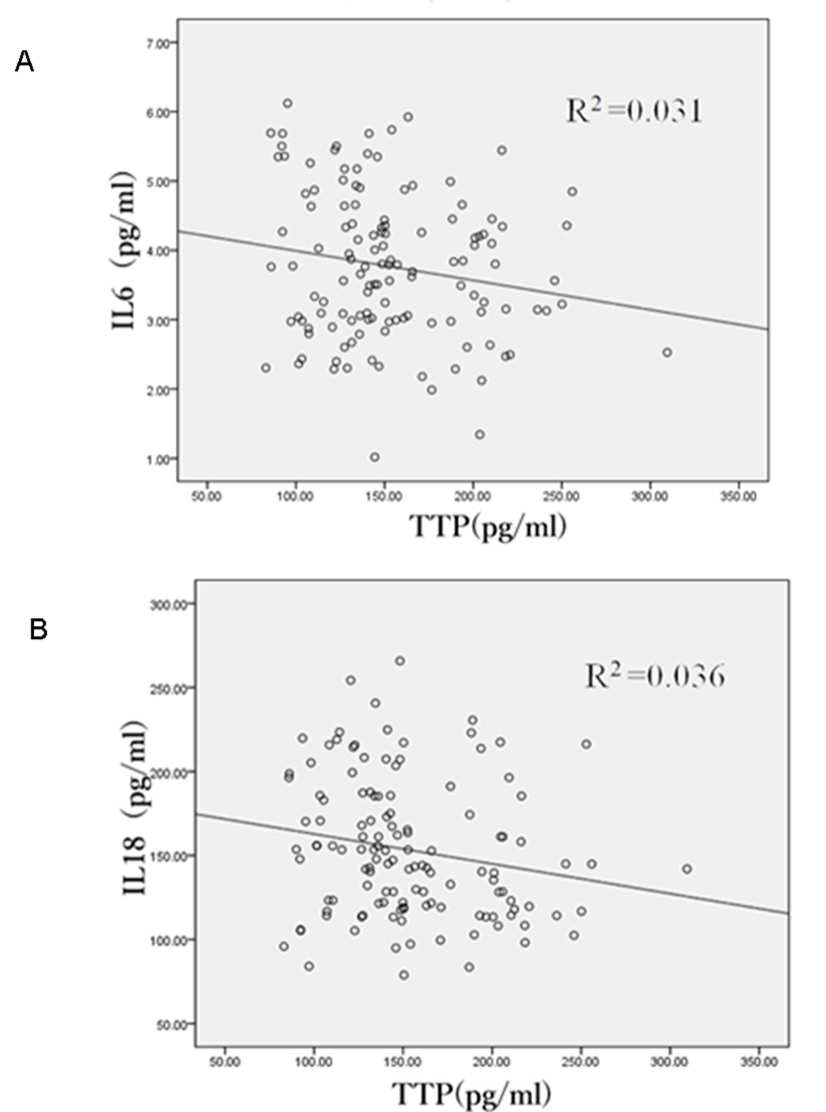
Inverse relationship between serum TTP and IL-18 or IL-6. The correlation between serum TTP and IL-6 (A) or IL-18 (B) protein levels was determined by plotting the corresponding values for individual diabetic patients. A best fit line is drawn.

To further explore the relationship between serum TTP and IL-6/IL-18 protein, we performed correlation analysis. This analysis revealed an inverse correlation in diabetic patients between serum level of TTP and the levels of IL-6 (correlation coefficient -0.572; R^2^ = 0.031; *P*<0.05) and IL-18 (correlation coefficient -0.685; R^2^ = 0.036; *P*<0.05) (Fig 3). These results are consistent with the possibility that TTP negatively regulates the expression of IL-6 and IL-18 in diabetes.

## Discussion

DN is the most common cause of end-stage kidney disease worldwide and accounts for a significant increase in morbidity and mortality in patients with diabetes. Early detection is critical in improving clinical management [7]. Although microalbuminuria is regarded as the gold standard for diagnosing the onset of DN, its predictive powers are limited. Thus, more sensitive and specific biomarkers are needed to improve the diagnostic capability of early stages of DN. Consequently, great efforts have been made in recent years to identify better strategies for the detection of early stages of DN and progressive kidney function decline in diabetic patients. Recently, a variety of omics and quantitative techniques in systems biology are rapidly emerging in the field of biomarker discovery, including proteomics, transcriptomics, and metabolomics, and they have been applied to search for novel putative biomarkers of diabetic nephropathy [8]. Novel biomarkers or their combination with microalbuminuria provide a better diagnostic accuracy than microalbuminuria alone, and may be useful for establishing personal medicine. Furthermore, the identification of novel biomarkers may provide insight into the mechanisms underlying DN.

We demonstrated an elevation of IL-6 and IL-18 mRNA and protein in serum and urine from T2DM patients, which supports the use of cytokines as markers reflecting the acceleration of microinflammation [9–11]. Additionally, we showed that TTP expression was reduced in patients with clinical proteinuria, with proportionately less reduction in diabetes patients with a reduced extent of DN. These results suggest that TTP levels may be more reflective of the microinflammatory state that occurs in diabetic patients prior to the occurrence of DN [12–14]. The present study is consistent with another study showing that IL-18 expression is associated with significantly worsened parameters of renal injury in T2DM patients. IL-18 is known to induce organ injury, and its relationship to kidney injury has been reported. Our observation that TTP is decreased in T2DM is consistent with the role of this protein as an inflammation inhibiting factor. The decrease in TTP was shown to precede the elevation of IL-18 [15,16]. Therefore, the reduction of TTP more closely indicates kidney injury than does IL-18 in T2DM. We also showed that the expression of TTP correlated inversely to the expression of both IL-6 and IL-18. This finding verifies the association of TTP with inflammatory factors.

Though the results presented are statistical, it should be pointed out that the sample size in our study was relatively small. This might the reason why a large spread was shown in the correlation analysis between serum TTP and IL-6/18. In the next study, more subjects will be recruited to confirm the results. In addition, as a cross-sectional study, it is difficult to know if there is any causality between the decrease in TTP levels and increase in interleukin levels in diabetes patients. In the next study with follow-up observation, TPP and interleukins will be measured and analyzed at multiple time points to ascertain if decrease of TTP levels is the cause of increase of interleukins in diabetes patients.

In summary, T2DM induces a significant increase of IL-6 and IL-18 production and a significant decrease of TTP. Reduced serum and urine TTP might precede the elevation of IL-6 and IL-18, which exacerbates kidney injury [17]. Therefore, our results suggest that TTP may more closely reflect T2DM than does IL-6 and IL-18. This implies that after these results are further confirmed by the next prospective studies, TTP might be considered as an early prognostic factor when the cut-off values of the serum and/or urinary TTP levels for diabetic proteinuria are calculated from enough representative diabetes patients, and potentially as a therapeutic target, for DN.
